# Supplementary figures and images for: DMC1 attenuates RAD51-mediated recombination in Arabidopsis
Source: PLoS Genet. 2022 Aug 25;18(8):e1010322. doi: 10.1371/journal.pgen.1010322 (PMC9451096; doi:10.1371/journal.pgen.1010322)

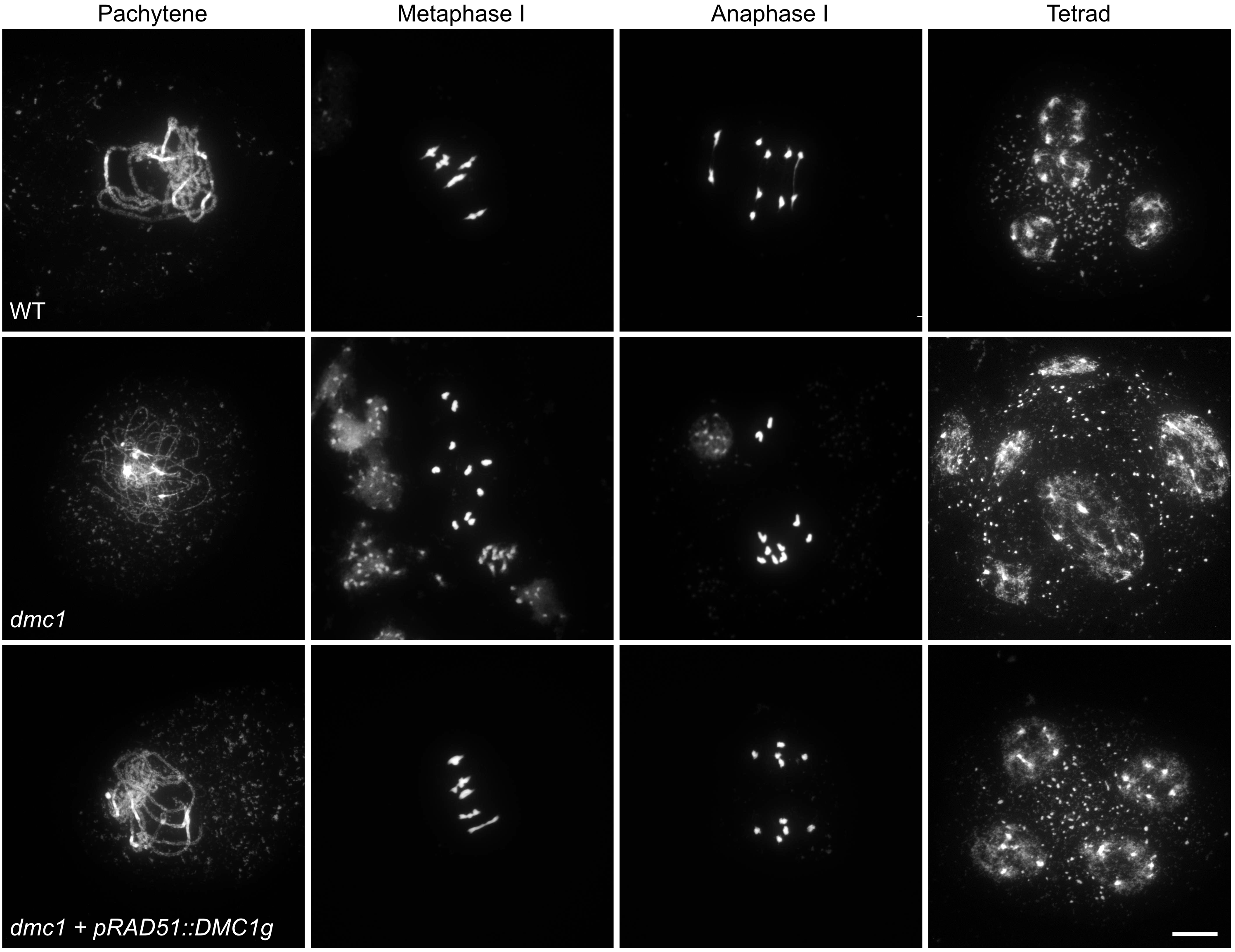

Supplement: S1 Fig — In WT meiosis, full pairing and synapsis is observed during Pachytene. Chromosomes condense and five bivalents linked by chiasmata are visualized during Metaphase I. Homologous chromosomes segregate in Anaphase I and Meiosis II proceeds leading to the formation of four balanced nuclei. In contrast, dmc1 mutants exhibit defects in pairing and synapsis in pachytene, and 10 univalents are visible at Metaphase I due to absence of chiasmata. Univalents segregate randomly in Anaphase I and this leads, after Meiosis II, to polyads. The meiotic defects of the dmc1 mutant are complemented by the presence of pRAD51::DMC1g. (Scale Bar: 10 μm). (TIFF) [file pgen.1010322.s001.tiff]

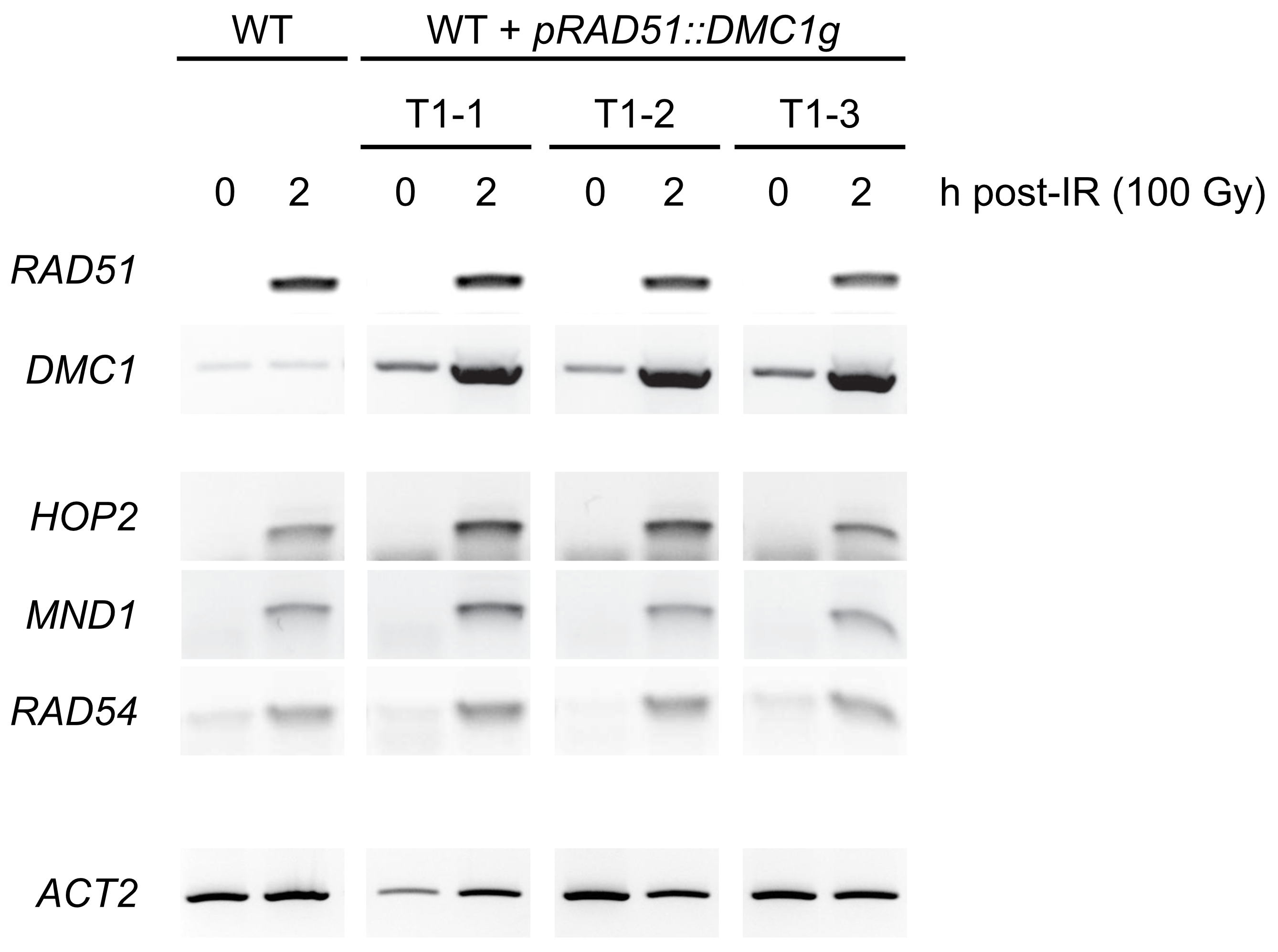

Supplement: S2 Fig — RT-PCR expression analysis of RAD51, DMC1, RAD54 and HOP2/MND1 in 7-day-old untreated or gamma-irradiated seedlings expressing or not the pRAD51::DMC1g transgene. Seedlings were irradiated at 100 Gy and expression was analyzed 2 hours after irradiation. Actin is used as a loading control. (TIFF) [file pgen.1010322.s002.tiff]

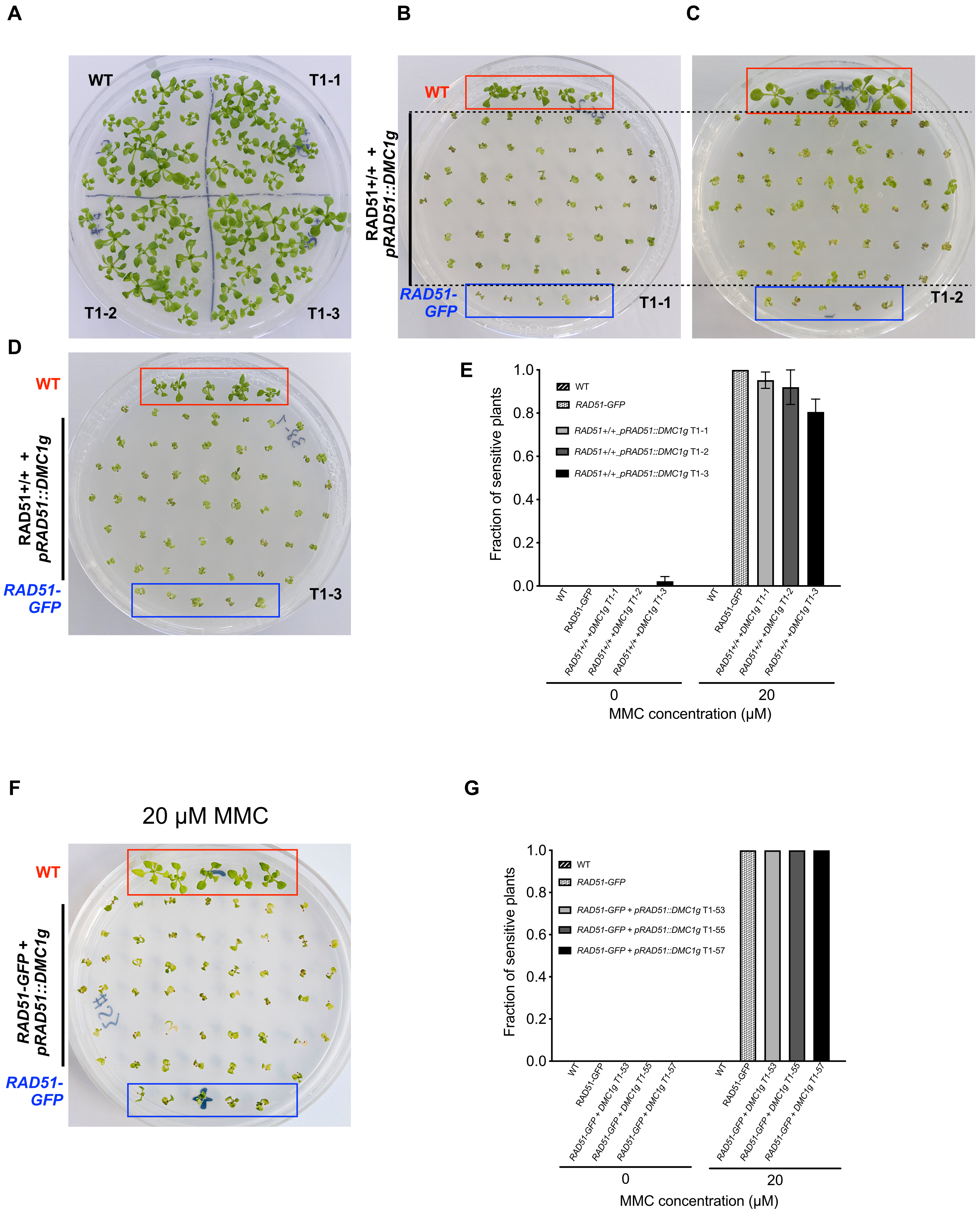

Supplement: S3 Fig — (A to E) Mitomycin C hypersensitivity of transgenic RAD51+/+ seedlings overexpressing DMC1. Pictures of two-week-old seedlings grown without (A) or with 20 μM MMC (B to D) are shown. (E) Sensitivity of the seedlings was scored after 2 weeks and the fractions of sensitive plants (plants with less than 4 true leaves) are shown (3 biological repeats, each with N > 45 seedlings). Three independent RAD51+/+ lines expressing the pRAD51::DMC1 transgene were tested (T1-1, T1-2 and T1-3). (F-G) MMC sensitivity of RAD51-GFP lines overexpressing DMC1. (F) Pictures of two-week-old seedlings grown with 20 μM MMC. (G) Sensitivity of the seedlings was scored after 2 weeks and the fractions of sensitive plants (plants with less than 4 true leaves) are shown. Three independent RAD51-GFP lines overexpressing DMC1 were tested (N > 45) and all three lines showed strong hypersensitivity to MMC. The RAD51-GFP fusion protein forms RAD51 filaments that support the activity of DMC1 in meiosis [39] but this is not sufficient for DMC1 mitotic activity. (TIFF) [file pgen.1010322.s003.tiff]

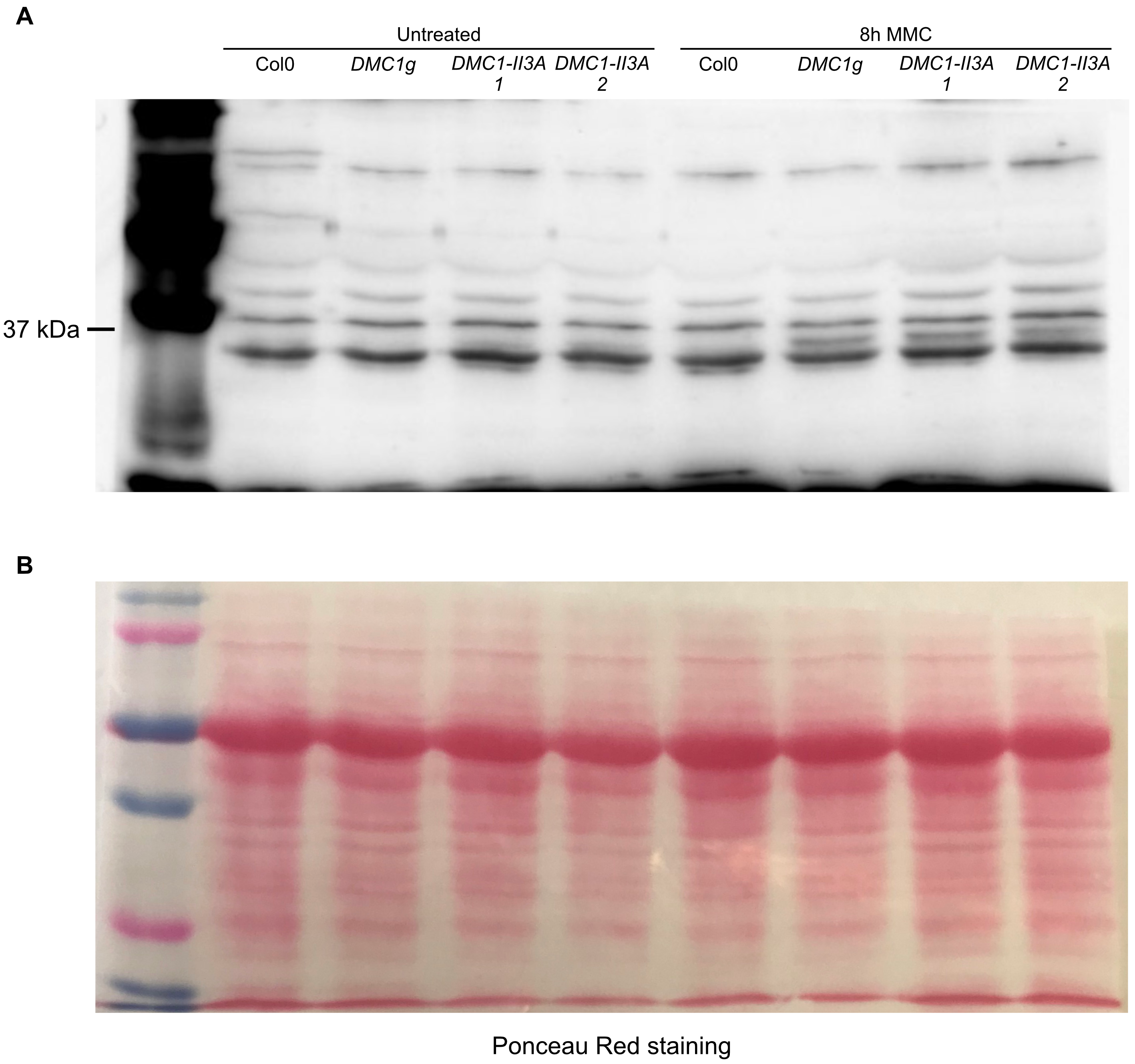

Supplement: S5 Fig — (A) DMC1g and DMC1-II3A protein are induced by MMC treatment. Total proteins were extracted from 1-week-old seedlings treated or not with 30 μM MMC for 8 hours and DMC1 abundance measured. No DMC1-specific band (37 kDa) is observed in untreated plants in both wild-type and transgenic plants. In contrast, while DMC1 is still absent in wild-type plants after DNA damage treatment, it becomes clearly visible in transgenic plants expressing either DMC1g or DMC1-II3A. (B) Ponceau Red staining of the above membrane before incubation with anti-DMC1 antibody, showing equal loading. (TIFF) [file pgen.1010322.s005.tiff]

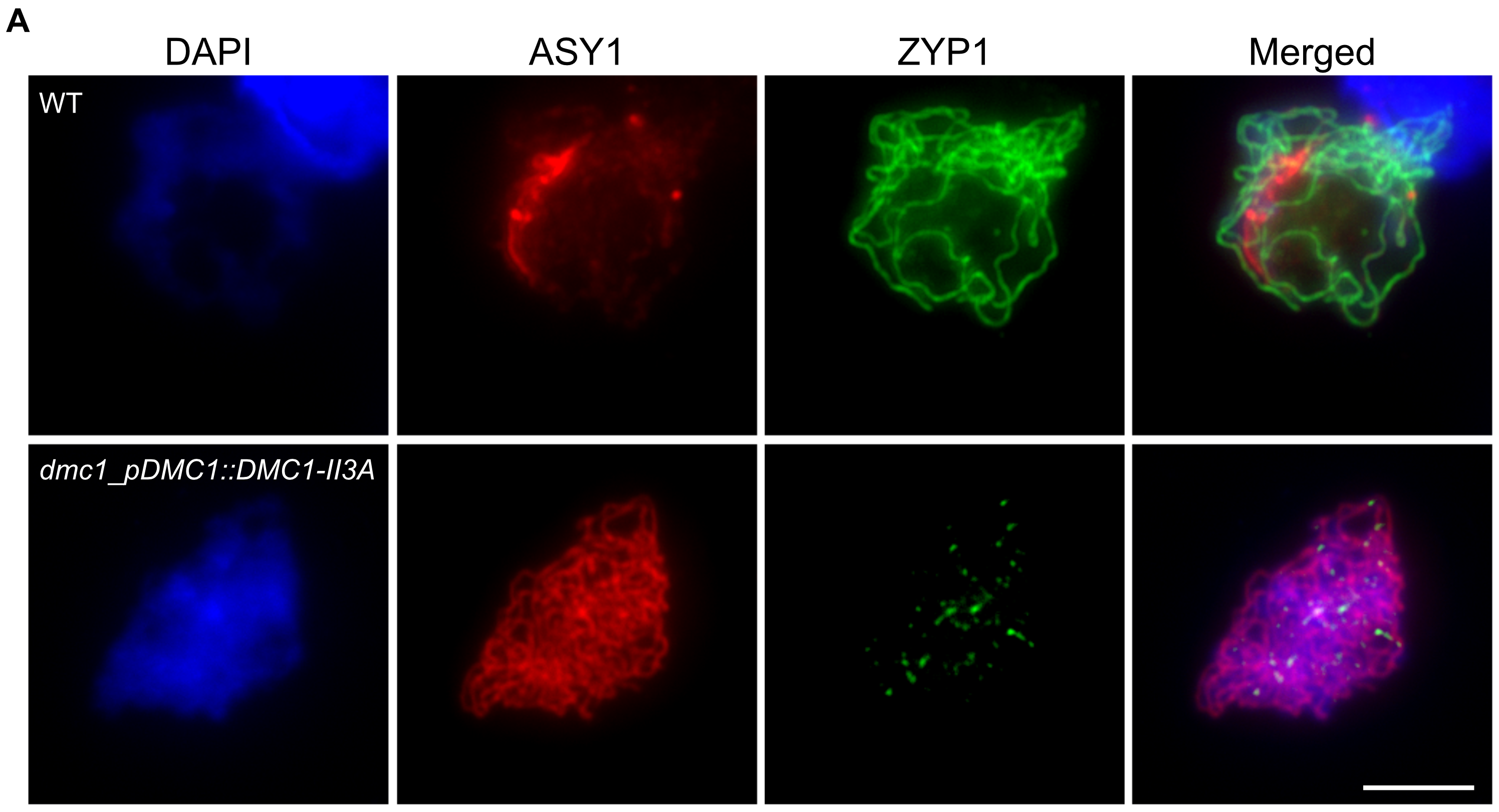

Supplement: S6 Fig — (A) Male meiocytes stained with the ASY1 antibody (red) and the ZYP1 antibody (green). In wild-type pachytene, ZYP1 extends along the entire length of the chromosomes. In dmc1 plants expressing DMC1-II3A, ZYP1 staining is restricted to a few foci and short stretches. (Scale bar: 5μm). (TIFF) [file pgen.1010322.s006.tiff]

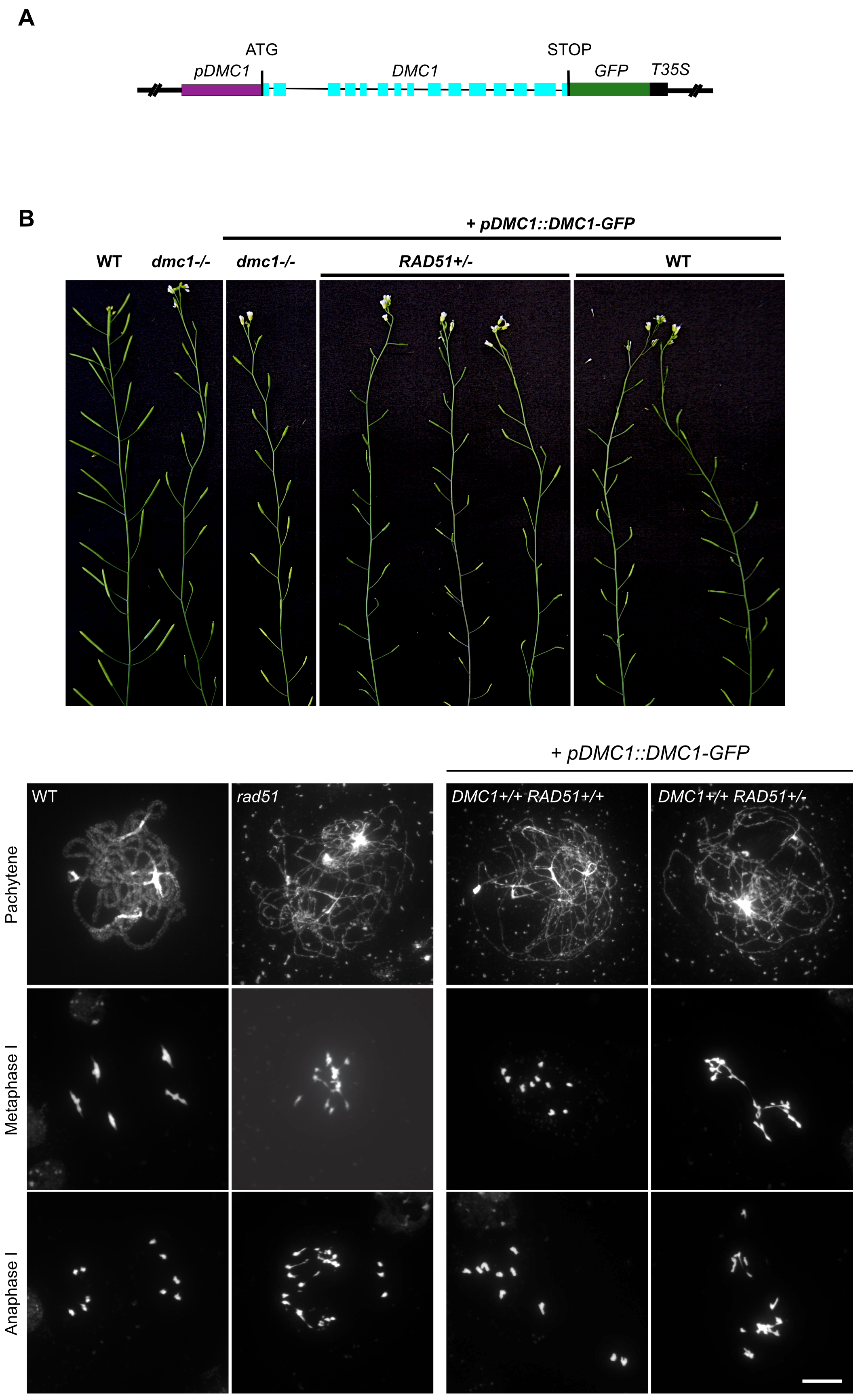

Supplement: S7 Fig — (A) Schematic representation of the pDMC1::DMC1-GFP construct. (B) Wild-type plants have long siliques, full of seeds, while dmc1 mutants are nearly sterile. Expression of pDMC1::DMC1-GFP in dmc1 mutants does not restore fertility and even strongly reduces fertility when expressed in rad51+/- or WT plants. (C) DAPI staining of chromosomes during meiosis. Wild-type cells show pairing and synapsis of homologous chromosomes in pachytene, five bivalents at metaphase I and two groups of five chromosomes at anaphase I. rad51 mutants exhibit defective synapsis and extensive chromosome fragmentation. Meiotic spreads in DMC1+/+ RAD51+/+ plants expressing DMC1-GFP revealed a dmc1-like phenotype with defective synapsis but ten intact univalents at metaphase I, which randomly segregate at anaphase I. In contrast, extensive fragmentation is observed in DMC1+/+ RAD51+/- plants expressing DMC1-GFP. (Scale Bar: 10 μm). (TIFF) [file pgen.1010322.s007.tiff]

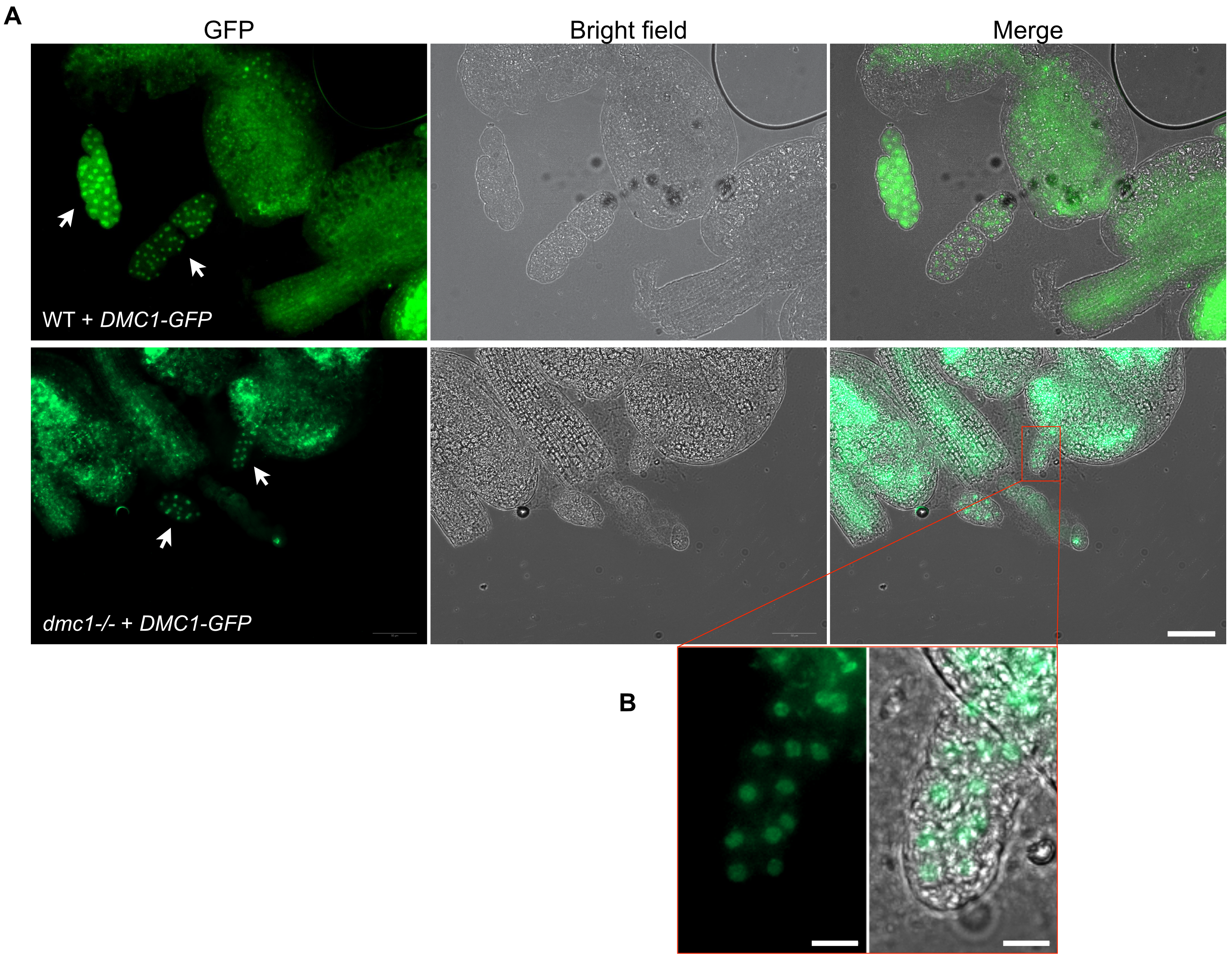

Supplement: S8 Fig — (A-B) In vivo observation of DMC1-GFP fluorescence in meiocytes. DMC1-GFP fluorescence is observed in nuclei of pDMC1::DMC1-GFP transgenic plants. (Scale bar = 50 μm in A and 10 μM for close-up view in B). (TIFF) [file pgen.1010322.s008.tiff]
