# Supplementary material for: DMC1 attenuates RAD51-mediated recombination in Arabidopsis
Source: PLoS Genet. 2022 Aug 25;18(8):e1010322. doi: 10.1371/journal.pgen.1010322 (PMC9451096; doi:10.1371/journal.pgen.1010322)
Supplement: S4 Fig — The alignment was generated using ClustalW. Numbers indicate amino acid positions. Red letters indicate the three conserved amino acids (R136A, R308A, K318A in Arabidopsis) that have been mutated in yeast to generated dmc1-II3A. Under the sequences, asterisks, colons and full stops indicate identical, conserved and semi-conserved residues respectively. (DOC) [file pgen.1010322.s004.doc]

ScDMC1 M--SVTGTEI---------DSDTAKNILS-VDELQNYGINASDLQKLKSGGIYTVNTVLS 48

AtDMC1-1 MMASLKAEETSQMQLVEREENDEDEDLFEMIDKLIAQGINAGDVKKLQEAGIHTCNGLMM 60

AtDMC1-II3A

* *:.. * :.* ::::. :*:* ****.*::**:..**:* * ::

ScDMC1 TTRRHLCKIKGLSEVKVEKIKEAAGKIIQVGFIPATVQLDIRQRVYSLSTGSKQLDSILG 108

AtDMC1-1 HTKKNLTGIKGLSEAKVDKICEAAEKIVNFGYMTGSDALIKRKSVVKITTGCQALDDLLG 120

AtDMC1-II3A

*:::* ******.**:** *** **::.*::..: * *: * .::**.: **.:**

ScDMC1 GGIMTMSITEVFGEF**R**CGKTQMSHTLCVTTQLPREMGGGEGKVAYIDTEGTFRPERIKQI 168

AtDMC1-1 GGIETSAITEAFGEF**R**SGKTQLAHTLCVTTQLPTNMKGGNGKVAYIDTEGTFRPDRIVPI 180

AtDMC1-II3A **A**

*** * :***.*****.****::********** :* **:**************:** *

ScDMC1 AEGYELDPESCLANVSYARALNSEHQMELVEQLGEELSSGDYRLIVVDSIMANFRVDYCG 228

AtDMC1-1 AERFGMDPGAVLDNIIYARAYTYEHQYNLLLGLAAKMSEEPFRILIVDSIIALFRVDFTG 240

AtDMC1-II3A

** : :** : * *: **** . *** :*: *. ::*. :*:::****:* ****: *

ScDMC1 RGELSERQQKLNQHLFKLNRLAEEFNVAVFLTNQVQSDPGASALFASADGRKPIGGHVLA 288

AtDMC1-1 RGELADRQQKLAQMLSRLIKIAEEFNVAVYMTNQVIADPGGG-MFIS-DPKKPAGGHVLA 298

AtDMC1-II3A

****::***** * * :* ::********::**** :***.. :* * * :** ******

ScDMC1 HASATRILL**R**KGRGDERVA**K**LQDSPDMPEKECVYVIGEKGITDSSD 334

AtDMC1-1 HAATIRLLF**R**KGKGDTRVC**K**VYDAPNLAEAEASFQITQGGIADAKD 344

AtDMC1-II3A **A A**

**:: *:*:***:** **.*: *:*::.* *. : * : **:*:.*

**S4 Fig.** Sequence alignment of DMC1 proteins from *Saccharomyces cerevisiae* (ScDMC1) and *Arabidopsis thaliana* (AtDMC1) and the corresponding mutated AtDMC1-II3A variant. The alignment was generated using ClustalW. Numbers indicate amino acid positions. Red letters indicate the three conserved amino acids (R136A, R308A, K318A in Arabidopsis) that have been mutated in yeast to generated *dmc1-II3A*. Under the sequences, asterisks, colons and full stops indicate identical, conserved and semi-conserved residues respectively.
