# Supplementary material for: DMC1 attenuates RAD51-mediated recombination in Arabidopsis
Source: PLoS Genet. 2022 Aug 25;18(8):e1010322. doi: 10.1371/journal.pgen.1010322 (PMC9451096; doi:10.1371/journal.pgen.1010322)
Supplement: S1 Table — (DOCX) [file pgen.1010322.s009.docx]

| Gene |  | Sequence 5' - 3' | Length (bp) |
| --- | --- | --- | --- |
|  |  |  |  |
| RAD51 | RT-PCR | GGCCATGTACATTGATGCTG | 248 |
|  |  | AAAGCTCTCCCCTTCCAGAG |  |
| DMC1 | RT-PCR | AAAGGAGGGAATGGAAAAGTG | 475 |
|  |  | GTGTATCGCCTTTGCCTTTC |  |
| HOP2 | RT-PCR | GAGAAACTGGTCAAACTACGT | 228 |
|  |  | ATATGCCTGGAAACTTAGACC |  |
| MND1 | RT-PCR | AGAACTTGTTGATCAGTGTGA | 213 |
|  |  | TTATCTGTCCATCTGTTAGCC |  |
| RAD54 | RT-PCR | CACTCCATTGGAAGAAGATCT | 158 |
|  |  | GACTGACATTTGATTCGATGG |  |
| ACTIN 2 | RT-PCR | CCTTGTACGCCAGTGGTCG | 662 |
|  |  | CTCGGCCTTGGAGATCCAC |  |
|  |  |  |  |

**S1 Table. Primers used in this study**
